# Supplementary material for: GPR4 Knockout Attenuates Intestinal Inflammation and Forestalls the Development of Colitis-Associated Colorectal Cancer in Murine Models
Source: Cancers (Basel). 2023 Oct 13;15(20):4974. doi: 10.3390/cancers15204974 (PMC10605520; doi:10.3390/cancers15204974)
Supplement: Supplementary file 1 [file cancers-15-04974-s001.zip › cancers-2664502-supplementary.pdf]

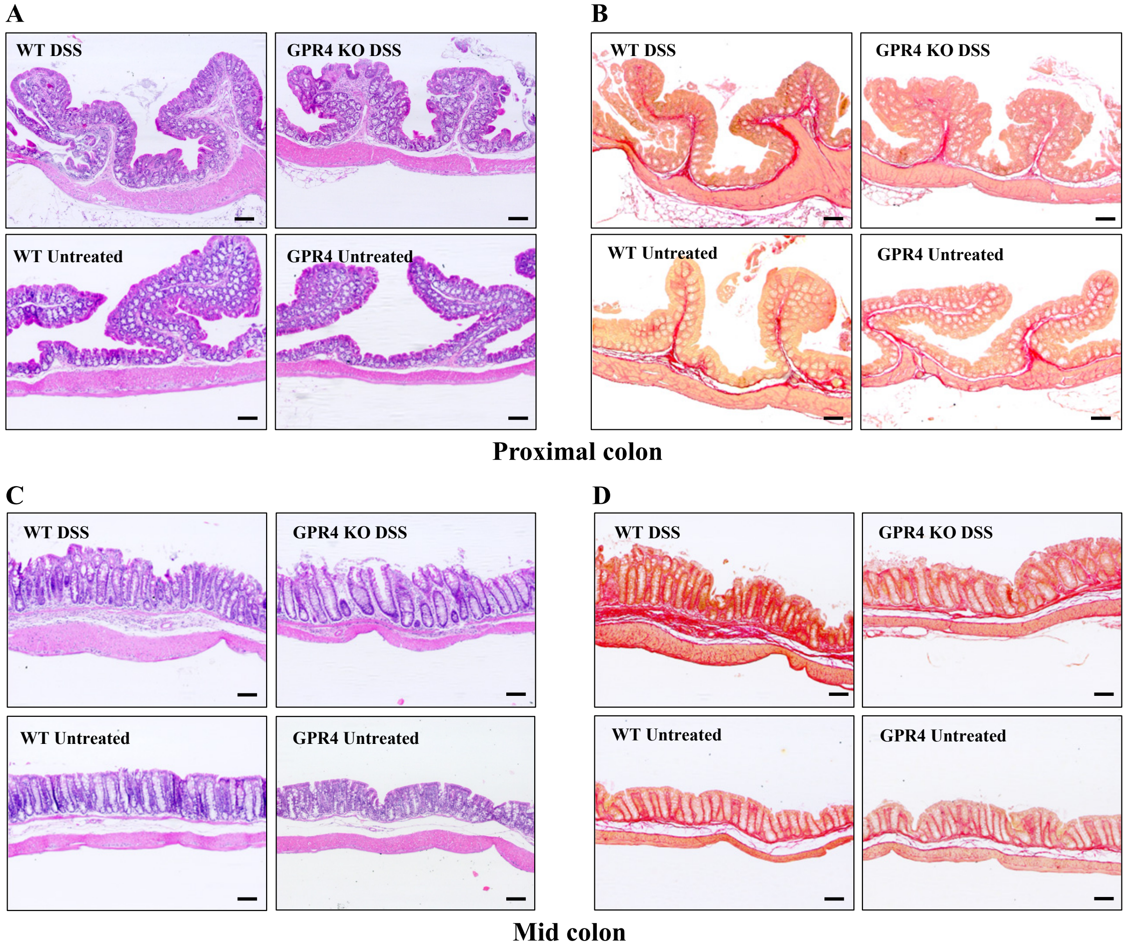

Supplementary Figure S1. Histology of proximal and middle colon in chronic colitis mice. (A) Representative H&E pictures of the proximal colon for WT-DSS, GPR4 KO-DSS, WT control, and GPR4 KO control mice. (B) Representative pictures of Picrosirius red stained tissue sections of the proximal colon for WT-DSS, GPR4 KO-DSS, WT control, and GPR4 KO control mice. (C) Representative H&E pictures of the middle colon for WT-DSS, GPR4 KO-DSS, WT control, and GPR4 KO control mice. (D) Representative pictures of Picrosirius red stained tissue sections of the middle colon for WT-DSS, GPR4 KO-DSS, WT control, and GPR4 KO control mice. Scale bar is 100µm.

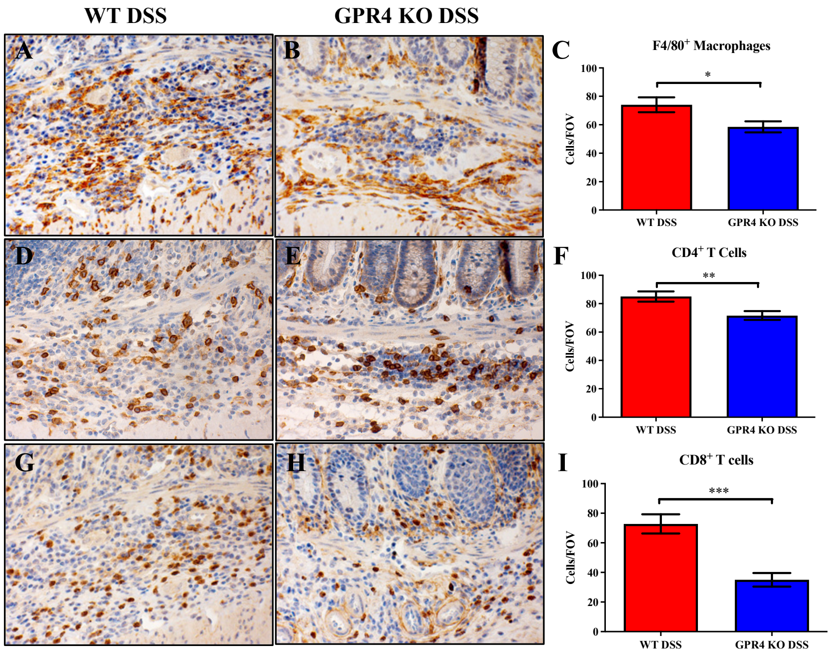

Supplementary Figure S2. Immune cell infiltration in the distal colon of chronic DSS mice. GPR4 deletion results in fewer immune cell infiltrates such as (A-C) F4/80+ macrophages, (D-F) CD4+ T cells, and (G-I) CD8+ T cells into the inflamed intestine. WT-DSS (n=5-6) and GPR4 KO-DSS (n=5-6). Data are presented as mean ± SEM and were analyzed for statistical significance using the unpaired *t*-test between WT-DSS mice and GPR4 KO-DSS mice. (**P* < 0.05, ***P* < 0.01, *** *P*< 0.001)

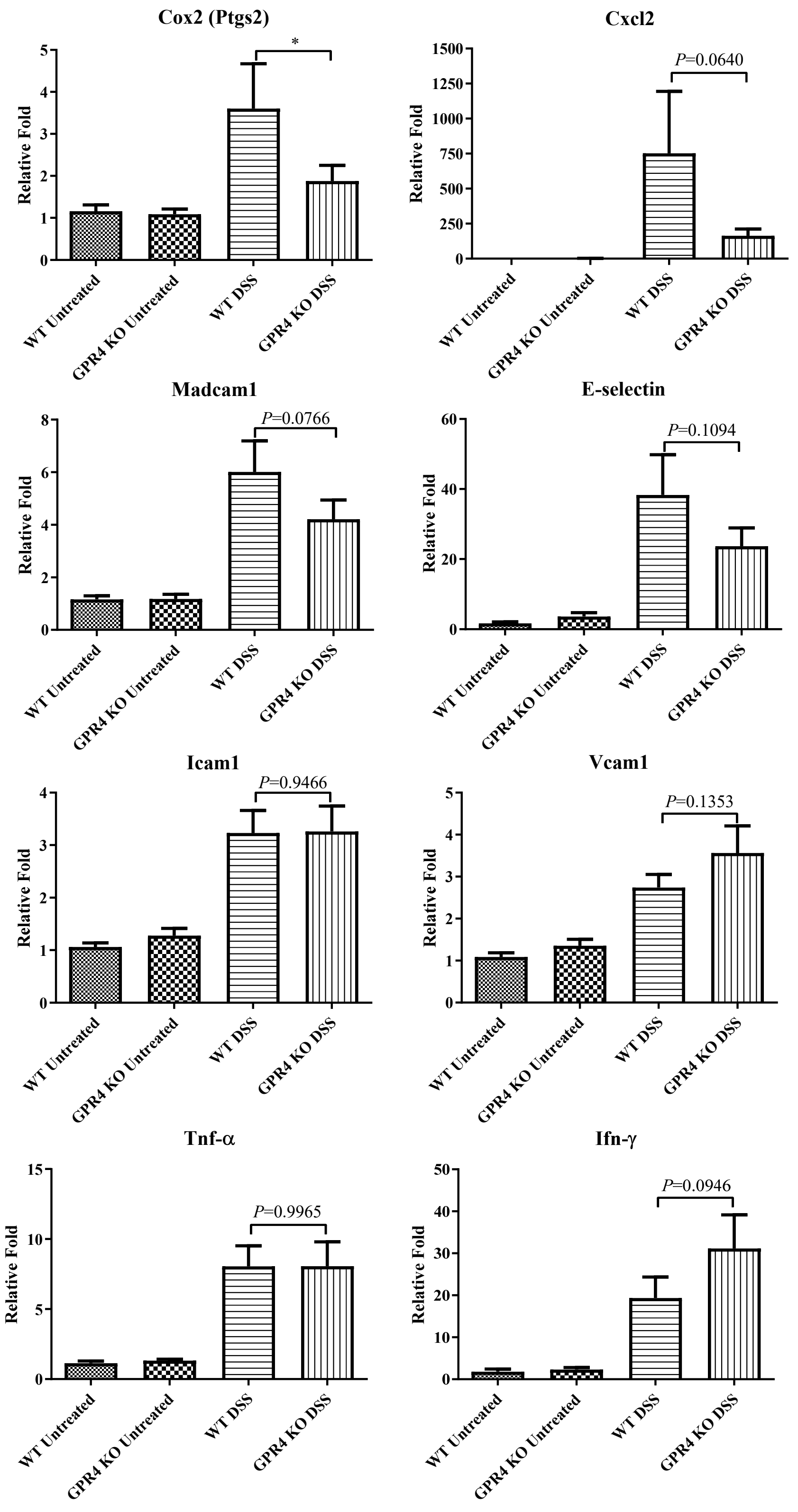

Supplementary Figure S3. Inflammatory gene expression in the colon of chronic DSS and control mice. Total RNA was isolated from mouse colon tissues and TaqMan qRT-PCR was performed to measure gene expression. WT control (n=18), GPR4 KO control (n=14), WT-DSS (n=17), and GPR4 KO-DSS (n=18). One-way ANOVA followed by Sidak test was used to compare the relative gene expression between the WT-DSS and GPR4 KO-DSS groups. (*$P < 0.05$)
